# Cardiac Macrophages and Their Effects on Arrhythmogenesis

**DOI:** 10.3389/fphys.2022.900094

**Published:** 2022-06-22

**Authors:** Ruibing Xia, Philipp Tomsits, Simone Loy, Zhihao Zhang, Valerie Pauly, Dominik Schüttler, Sebastian Clauss

**Affiliations:** ^1^ Department of Medicine I, University Hospital Munich, Ludwig-Maximilians-University Munich (LMU), Munich, Germany; ^2^ Institute of Surgical Research at the Walter-Brendel-Centre of Experimental Medicine, University Hospital Munich, Ludwig-Maximilians-University Munich (LMU), Munich, Germany; ^3^ German Centre for Cardiovascular Research (DZHK), Partner Site Munich, Munich Heart Alliance, Munich, Germany

**Keywords:** Cardiac electrophysiogy, macrophages, arrhythmia, inflammation, animal models, translational medicine

## Abstract

Cardiac electrophysiology is a complex system established by a plethora of inward and outward ion currents in cardiomyocytes generating and conducting electrical signals in the heart. However, not only cardiomyocytes but also other cell types can modulate the heart rhythm. Recently, cardiac macrophages were demonstrated as important players in both electrophysiology and arrhythmogenesis. Cardiac macrophages are a heterogeneous group of immune cells including resident macrophages derived from embryonic and fetal precursors and recruited macrophages derived from circulating monocytes from the bone marrow. Recent studies suggest antiarrhythmic as well as proarrhythmic effects of cardiac macrophages. The proposed mechanisms of how cardiac macrophages affect electrophysiology vary and include both direct and indirect interactions with other cardiac cells. In this review, we provide an overview of the different subsets of macrophages in the heart and their possible interactions with cardiomyocytes under both physiologic conditions and heart disease. Furthermore, we elucidate similarities and differences between human, murine and porcine cardiac macrophages, thus providing detailed information for researchers investigating cardiac macrophages in important animal species for electrophysiologic research. Finally, we discuss the pros and cons of mice and pigs to investigate the role of cardiac macrophages in arrhythmogenesis from a translational perspective.

## 1 Introduction

Cardiac arrhythmias affect millions of individuals worldwide and are a major cause of morbidity and mortality, thereby causing a significant medical but also socioeconomic burden (Boriani et al., 2006; Bloch Thomsen et al., 2010; Gorenek et al., 2014). The pathophysiology of arrhythmias is complex and includes alterations of electrical, structural, and contractile properties of the heart as well as changes of autonomic cardiac innervation (Nattel et al., 2020). Cardiac inflammatory responses e.g., in the context of myocardial infarction (MI) or heart failure (HF), are thought to crucially contribute to the proarrhythmic remodeling resulting in the development of vulnerable substrates in the heart (Engelmann and Svendsen, 2005; Francis Stuart et al., 2016). Recently, studies have revealed the various roles played by immune cells, especially macrophages, in the maintenance of cardiac homeostasis (Hulsmans et al., 2017; Sugita et al., 2021; Simon-Chica et al., 2022) and—if disrupted—the development of cardiac arrhythmias (Monnerat et al., 2016; Sun et al., 2016; Yin et al., 2016; Fei et al., 2019; Hu et al., 2019; Liu et al., 2019; Lubos et al., 2020; Lyu et al., 2020; Miyosawa et al., 2020; Zhang et al., 2020; Hiram et al., 2021; Zhang et al., 2021).

Being a key component of the innate immunity, cardiac macrophages reside in the heart from the embryonic stage and unfold significant roles during heart development (Epelman et al., 2014). In these early stages, macrophages from the embryonic yolk-sac or the fetal liver migrate into the heart where they continue to proliferate locally, thus establishing the pool of “resident cardiac macrophages,” which can be identified by a characteristic combination of surface markers such as CD45^+^CX_3_CR1^+^F4/80^high^ (Schulz et al., 2012; Molawi et al., 2014). After birth, bone marrow (BM)-derived monocytes circulate in the blood and—especially under pathologic conditions—migrate into the heart where they develop to macrophages (Epelman et al., 2014; Heidt et al., 2014). Due to their different origin these can be called “recruited cardiac macrophages” and can be identified as CD11b^high^F4/80^low^.

The majority of research focused on these BM-derived “recruited macrophages”, which can be grouped according to their functional phenotype into classic inflammatory and noncanonical reparative macrophages (Mills, 2012; Lavine et al., 2014). The exact identities of these macrophages remain undefined. Pro-inflammatory macrophages release cytokines and chemokines and have been demonstrated to be involved in electrical (Monnerat et al., 2016; Sun et al., 2016; Fei et al., 2019; Liu et al., 2019; Zhang et al., 2020) as well as autonomic remodeling of the heart (Yin et al., 2016; Hu et al., 2019; Lyu et al., 2020; Zhang et al., 2021). Noncanonical reparative (or alternatively-activated) macrophages release anti-inflammatory cytokines, participate in post-inflammatory tissue repair, and contribute to cardiac structural remodeling by promoting cardiac fibrosis (Wynn and Barron, 2010; Dobaczewski et al., 2011; Mills, 2012; Liao et al., 2018; Miyosawa et al., 2020; Watson et al., 2020; Hiram et al., 2021; Revelo et al., 2021). Furthermore, it has been shown that cardiac resident macrophages are functionally connected to cardiomyocytes by gap junctions (Simon-Chica et al., 2022), thereby facilitating the physiologic electrical conduction in the atrioventricular (AV) node (Hulsmans et al., 2017) or preserving electrical conduction in the context of disease (Sugita et al., 2021).

In sum, a growing body of evidence suggests a key role of cardiac macrophage populations in regulating both physiologic electrical conduction in the healthy heart and proarrhythmic remodeling processes in the diseased heart. With this review, we provide a comprehensive overview on cardiac macrophages and their effects on cardiac electrophysiology. We describe different macrophage populations in the heart and we summarize recent findings on how they affect regular electrophysiology and proarrhythmic electrical, structural, and autonomic remodeling. Since translating basic scientific findings into clinical practice is one of the major challenges in modern medicine, we summarize and discuss the current knowledge on cardiac macrophage populations in mice, pigs, and humans to support translational research on cardiac macrophages in the future.

## 2 Cardiac Macrophage Subpopulations

Macrophages are the most abundant leukocytes in the heart (Pinto et al., 2016; Skelly et al., 2018). They are highly plastic, play significant roles both in cardiovascular homeostasis and pathophysiology (Gomez et al., 2018) and can be identified by certain markers expressed on the cell surface.

Most knowledge about cardiac macrophage subpopulations is derived from studies in mice: during development, macrophages from the embryonic yolk-sac (at embryonic day (E) 7.25) and the fetal liver (at E8.25) migrate into the heart where they persist as “resident cardiac macrophages” by *in situ* proliferation (Schulz et al., 2012; Hashimoto et al., 2013). In the healthy heart, these resident macrophages are the predominant macrophage population, while in the diseased heart so called “recruited macrophages” derived from infiltrating bone-marrow monocytes (from E17.5 to adulthood) are the dominant population (Ingersoll et al., 2011; Epelman et al., 2014; Dick et al., 2019). Moreover, a recent report indicated the endocardium as an additional source of resident cardiac macrophages, called endocardial-derived macrophages, which emerge at E9.5, exhibit an intensive phagocytic activity, then proliferate *in situ*, and are indispensable for development and remodeling of extracellular matrix (ECM) and heart valves (Zhang et al., 2018; Shigeta et al., 2019).

In steady-state, resident macrophages are exclusively (CCR2^−^MHC-II^low^) or partly (CCR2^−^MHC-II^high^) replenished through *in situ* proliferation with negligible monocyte input (Dick et al., 2019). *In situ* proliferation also contributes to the macrophage proliferation driven by the interleukin (IL)-4 triggered inflammatory response (Jenkins et al., 2011). However, if resident macrophages are depleted (e.g. in transgenic mouse models), monocytes from the bone marrow and splenic reservoirs repopulate the heart and differentiate to macrophages (Heidt et al., 2014). Murine monocytes can be grouped into two major functionally distinct subsets: inflammatory Ly6C^high^ (CCR2^+^CX_3_CR1^low^Gr1^+^) and noninflammatory Ly6C^low^ (CCR2^−^CX_3_CR1^high^Gr1^-^) monocytes (Geissmann et al., 2003). All these different blood monocyte subsets differentiate into macrophages when stimulated with macrophage colony stimulating factor (M-CSF) *in vitro* (Sunderkotter et al., 2004), but in response to inflammatory stimuli bone marrow-derived Ly6C^high^ monocytes preferentially infiltrate inflammatory sites and differentiate into Ly6C^high^F4/80^high^ macrophages as demonstrated in a myocardial infarction (MI) mouse model (Nahrendorf et al., 2007).

Recruited and resident macrophages have different functions in the context of cardiac inflammation (Bajpai et al., 2019). Monocyte-derived macrophage subsets (CCR2^+^MHC-II^high^, CCR2^+^MHC-II^low^ and CCR2^+^Ly6C^high^) are preferentially recruited to sites of injury, where they enhance inflammation by promoting further monocyte and neutrophil recruitment and cause cardiomyocyte hypertrophy, interstitial fibrosis and adverse cardiac remodeling resulting in poor outcome, e.g. after myocardial infarction (Lavine et al., 2014; Li et al., 2016; Lorchner et al., 2018). In contrast, resident macrophage subsets (CCR2^−^MHC-II^low^Ly6C^low^ and CCR2^−^MHC-II^high^Ly6C^low^) demonstrate robust proangiogenic properties, suppress inflammation by inhibiting monocyte recruitment and enhance cardiac repair (Kaikita et al., 2004; Lavine et al., 2014; Bajpai et al., 2019). Profibrotic monocyte-derived MHC-II^high^ macrophages accumulate in mouse hearts exposed to aldosterone, activate fibroblasts by releasing IL-10 and transforming growth factor-β1 (TGF-β1), thereby causing collagen deposition and diastolic dysfunction (Hulsmans et al., 2018). In contrast, MHC-II^low^ macrophages favor the clearance of excess collagen by activating *in vivo* protease sensors and matrix metalloproteinases (MMPs) activity (Hulsmans et al., 2018).

Single-cell RNA sequencing (scRNA-seq) allows precise identification of macrophage subpopulations in the heart. In heart failure mice, two CCR2^−^ populations in the heart showed diverse functions: The MHC-II^high^CD163^+^Mrc1^+^ population is associated with antigen processing and shows a repair-mediating identity, while the other MHC-II^low^ population mediates phagocytosis and proinflammatory changes. The latter CCR2^−^MHC-II^low^ population can further be subdivided into two subclusters based on the cells’ different expresses level of the repair-associated genes *Mmp9* and *Arg2* and the neutrophil-associated gene *Csf3r*. On the other hand, two CCR2^+^ populations highly express the proinflammatory cytokines oncostatin M (Osm) and IL-1β. Intriguingly, overexpression of Osm was also observed in cardiac patient hearts suggesting a role in heart disease (Gautier et al., 2012; Skelly et al., 2018; Martini et al., 2019). scRNA-seq data indicates that human and murine TLF^+^ (FOLR2^+^ and/or TIMD4^+^ and/or LYVE1^+^) macrophages originate from both yolk sac and fetal monocyte precursors, and are the most transcriptionally conserved subset (Dick et al., 2022). In the adult human heart, LYVE1^+^TIMD4^−^ macrophages appear to be related to resident macrophages and are associated with cardiovascular remodeling. LYVE1^+^FOLR2^+^ macrophages are monocyte-derived and express the chemoattractant cytokine genes *CCL13* and *CCL18*. Patients with dilated cardiomyopathy showed reduced numbers of LYVE1^+^ resident macrophages (subsets expressed *FOLR2* and *HSPH1*) and an increased number of inflammatory macrophages (subsets expressed *TREM2*, *CCL3* and *KLF2*) (Koenig et al., 2022). In contrast, LYVE1^−^FOLR2^−^MERTK^-^ macrophages are associated with antigen presentation (Litvinukova et al., 2020).

In addition to the classification based on their origin, macrophages can be categorized depending on their cytokine secretion profile studied *in vitro* (Murray and Wynn, 2011). Commonly, macrophages producing proinflammatory cytokines such as IL-1β and tumor necrosis factor-α (TNF-α) which mediate pathogen clearance and tissue destruction, are defined as M1 macrophages. In contrast, macrophages releasing anti-inflammatory cytokines such as IL-10 and TGF-β1 contribute to healing and tissue repair and are commonly referred to as M2 macrophages (Chavez-Galan et al., 2015). Of note, the M1/M2 classification, while useful, is an oversimplification and applies to *in vitro* polarized macrophages, and should therefore be used with caution, especially when discussing *in vivo* macrophage phenotypes.

Generally, all kinds of inflammation contain a mixture of M1 and M2 responses (Atri et al., 2018). For example, in an infectious myocarditis mouse model, M1 macrophages which are activated by cytokines such as interferon-γ produced by T helper cells (Th1), support polarization of CD4^+^ T cells to produce IL-12, IL-23 and nitric oxide (NO). In addition, T helper cell (Th2)-produced cytokines such as IL-4 and IL-13, activate M2 macrophages, which control the proliferation of T cells and attenuate the immune response demonstrating that pro- and anti-inflammatory processes of cardiac macrophages happen simultaneously and disrupt homeostasis (Mills et al., 2000; Gutierrez et al., 2014). Furthermore, within this M1/M2 classification, different macrophage phenotypes partly express similar surface markers, and the exact lineage relationship of Ly6C^high^ and Ly6C^low^ monocytes to M1 and M2 macrophages has yet to be determined. In the acute phase of a myocardial infarction, Ly6C^high^ monocyte-derived macrophages are activated and generate proinflammatory cytokines, remove debris and apoptotic neutrophils, and contribute to tissue-rebuilding post-MI, a process to which Ly6C^low^ macrophages also contribute by regulating wound healing, angiogenesis and myofibroblast activation (Nahrendorf et al., 2007; Frantz and Nahrendorf, 2014).

In sum, there are several macrophage subpopulations in the heart that can be grouped by their origin, their functional impact, or the cytokines produced. However, there are significant overlaps between groups and several surface markers are not exclusively expressed by only one specific macrophage subset. In this review, we try to follow an origin-oriented classification of macrophages into embryonic/fetal precursor-derived “resident cardiac macrophages” and bone marrow monocyte-derived “recruited cardiac macrophages” wherever possible.

## 3 Cardiac Electrophysiology and Potential Arrhythmia Mechanisms

Studying the effects of immune cells on arrhythmogenesis requires profound knowledge about normal electrophysiology, especially regarding cardiac ion currents, calcium homeostasis, and cell-to-cell contacts. For an in-depth overview on cardiac ion channels and electrophysiology, the interested reader may be referred to specific reviews (Bosch, 2002; Bartos et al., 2015; Landstrom et al., 2017; Kistamas et al., 2020). For context, we provide a brief overview on fundamental concepts in the following paragraphs.

### 3.1 Cardiac Ion Channels and Action Potentials

**FIGURE 1:**
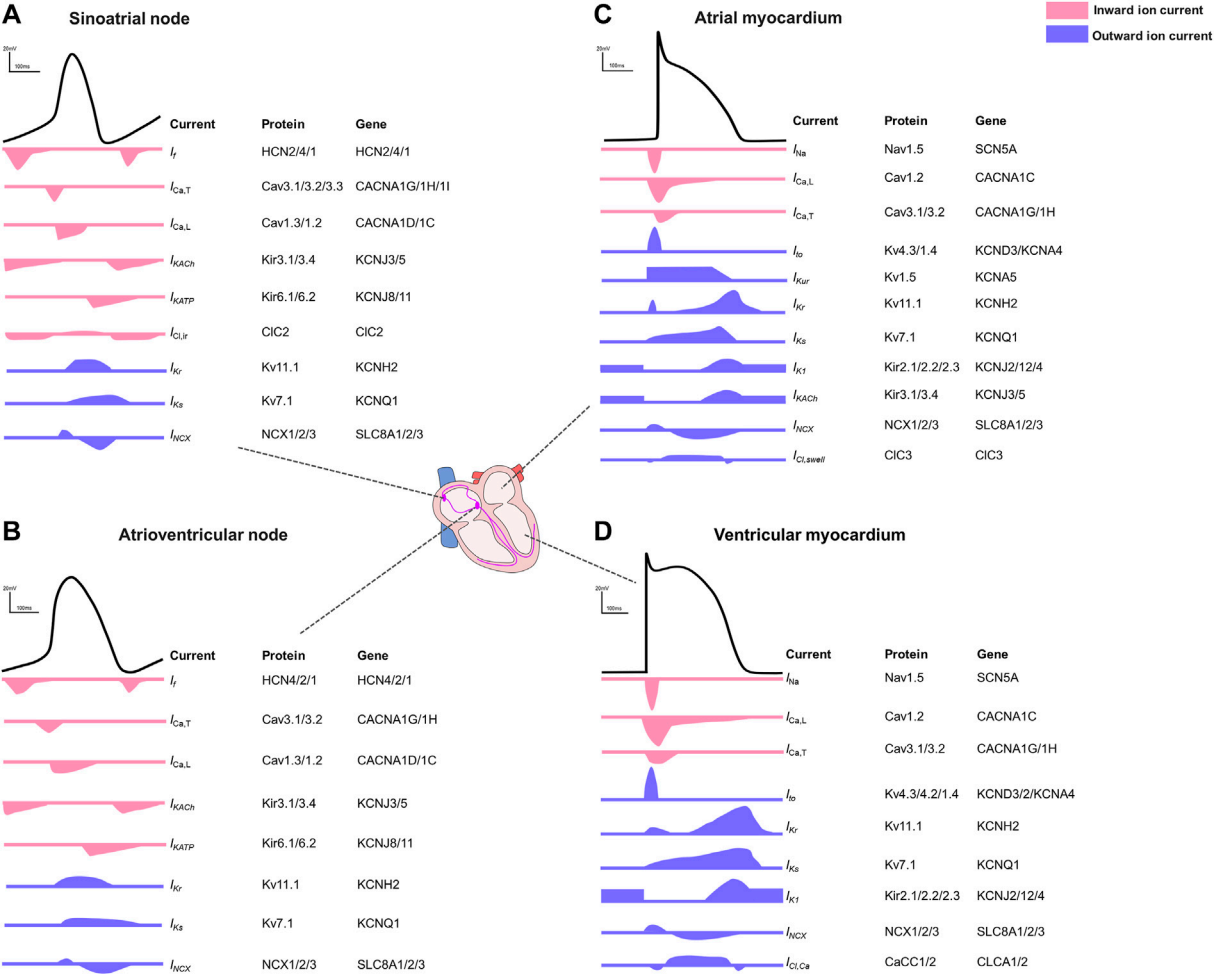
Action potentials and the contributing ion currents in different regions of the human heart. **(A)**. Sinoatrial node. **(B)**. Atrioventricular node. **(C)**. Atrial myocardium. **(D)**. Ventricular myocardium.

Myocardial electrical activity is generated by sequential opening and closing of ion channels and transporters establishing a transmembrane action potential (AP) in individual cardiomyocytes (Figure 1) (Nerbonne and Kass, 2005). The duration of the action potential (APD) reflects the interplay of inward and outward currents (Grant, 2009). In general, the cardiac action potential is divided into five phases: phase 0 reflects a rapid upstroke, which is generated by the activation of a fast inward Na^+^ current (*I*_Na_). Immediately following the AP upstroke, a transient repolarization produced by an outward potassium current (*I*_to_) follows (phase 1). Phase 2 represents a slowly decaying plateau, resulting from an equilibrium of the delayed-rectifier potassium current with slow, rapid, and ultrarapid activation kinetics (*I*_Ks_, *I*_Kr_, and *I*_Kur_, respectively), the Na^+^-K^+^-ATPase current (*I*_NKA_), the late sodium current (*I*_Na,Late_) and a simultaneous prominent calcium influx (*I*_Ca,L_, *I*_Ca,T_). Phase 3 is characterized by a rapid repolarization, resulting from the inactivation of calcium channels and the activation of an inward rectifier potassium current (*I*_K1_). The resting state (phase 4), which is also mainly driven by the potassium current *I*_K1_ establishes the resting membrane potential (RMP). Under physiologic conditions, the APD determines the effective refractory period (ERP), which is defined as the shortest time interval needed before a new stimulus can depolarize the cell again causing another AP (Nerbonne and Kass, 2005; Grant, 2009; Varro et al., 2021).

The electrophysiological properties illustrated by different AP shapes and durations differ between the different regions within the heart (Bartos et al., 2015) and between species (Clauss et al., 2019). In pacemaker cells of the sinoatrial node (SAN) or the atrioventricular node (AVN), the pacemaker current (*I*_f_) is responsible for the membrane hyperpolarization (Aziz et al., 2018). Inward Ca^2+^ currents, regulated mainly by the L-type calcium channel (*I*_Ca,L_, Ca_v_1.2), play a fundamental role in both depolarization of SAN and AVN cells and in counteracting repolarization despite lacking a clear plateau phase (Bers, 2008). Acetylcholine-activated K⁺ channels (*I*_K,Ach_ current) are most abundantly expressed in SAN and AVN where this current contributes to the diastolic depolarization and in the atria where it hyperpolarizes the cell contributing to the phase 3 repolarization (Nerbonne, 2016). In general, atrial action potentials are shorter than those in ventricles with a less clear plateau phase, mainly because of potassium channels with faster activation kinetics and larger conductance. In phase 4, the ventricular resting membrane potential is more negative because ventricular myocytes have higher inward rectifier currents than atrial myocytes (Ng et al., 2010). The regional differences driven by the diversity of ion channels (Joukar, 2021; Varro et al., 2021) are summarized in Figure 1.

### 3.2 Arrhythmia Mechanisms

Current paradigms in arrhythmogenesis include electrical ectopy (acting as a trigger) and reentry (acting as substrate) which are reviewed in detail elsewhere (Nattel et al., 2007; Michael et al., 2009; Wakili et al., 2011; Sanchez-Quintana et al., 2012; Clauss et al., 2015). Here, we briefly summarize some of the main aspects to allow a better understanding of macrophage effects on arrhythmogenesis which are provided in section 4.

#### 3.2.1 Ectopy/Arrhythmia Triggers

Any change to the subtle equilibrium of currents underlying cell type-specific action potentials (Figure 1) resulting from altered channel conduction or kinetics (e.g., by mutation, ligand binding or posttranslational modification) can potentially lead to focal ectopic activity which may in turn act as arrhythmia “trigger”. The main mechanisms underlying ectopic impulse generation include early (EADs) and delayed afterdepolarizations (DADs) which are voltage oscillations known to cause cardiac arrhythmias (Song et al., 2015). EADs appear at phase 2 (in the context of APD prolongation due to e.g., activation of L-type Ca^2+^ channels (*I*_Ca,L_ current)) or phase 3 (in the context of Ca^2+^ overload and activated Na^+^/Ca^2+^ exchange current (NCX) resulting in Na^+^ influx) (Tse, 2016). DADs usually occur following AP repolarization and are associated with an intracellular Ca^2+^ accumulation (Pogwizd et al., 2001).

#### 3.2.2 Reentry/Arrhythmia Substrate

Aside from altered impulse formation, abnormal impulse conduction represents another main mechanism of arrhythmogenesis (Nguyen et al., 2017). This can be caused by anatomic changes (such as hypertrophy or fibrosis) indirectly influencing conduction, by altered electrical conduction properties itself (functional reentry, e.g., by modified gap junction expression/function) (Kamjoo et al., 1997) as well as by changes in autonomic tone (Verheule and Schotten, 2021).

The major hallmark of structural remodeling and subsequently maintenance of arrhythmia is cardiac fibrosis, characterized by an imbalance between the generation and degradation of extracellular matrix (ECM) (Merchant and Armoundas, 2012). Under myocardial injury, activated fibroblasts differentiate into myofibroblasts, which release profibrotic factors such as transforming growth factor-β1 (TGF-β1) and stimulate expression of ECM, thereby promoting fibrogenesis (Khalil et al., 2017). Interstitial fibrosis forms electrical barriers between myocardial bundles, causing the electrical impulse to travel a longer distance, resulting in conduction delay and potential asynchronous activation of different areas of the myocardium (de Jong et al., 2011). Such a delayed impulse conduction, which also happens after myocardial infarction where a myocardial scar acts as a central obstacle, allows the myocardium to recover from a previous excitation. The following depolarizing front can continuously encounter excitable myocardium, thereby promoting circular excitation, so-called reentry (Verheule and Schotten, 2021). A murine TGF-β1 overexpression model for example shows that atrial fibrosis itself is sufficient to cause AF (Verheule et al., 2004). In ventricular myocardium, the slow conduction zone formed by fibrosis in post-MI and advanced HF patients creates a substrate for reentry of sustained monomorphic ventricular tachycardia (VT) (St John Sutton et al., 2003; Yokokawa et al., 2009).

The AP propagation depends on gap junctions, transmembrane proteins that mediate cell-to-cell coupling (Kanno and Saffitz, 2001). Gap junctions are composed of connexin (Cx) subunits. Cx43 is the most abundant connexin expressed in both atrial and ventricular cardiomyocytes of mammalian species (Severs et al., 2004). In healthy hearts, gap junctions are predominantly expressed in the intercalated disc regions between cardiomyocytes and facilitate the longitudinal flow of electrical currents (Rohr, 2004). Downregulated expression and lateralization of Cx43 increase arrhythmia susceptibility, as slowed and multidirectional conduction occurs (Kostin et al., 2004). Ischemia induced dephosphorylation and progressive reduction of Cx43 can lead to electrical uncoupling of ventricular myocytes, potentially playing an important role in arrhythmogenesis after MI (Beardslee et al., 2000).

Cardiac electrophysiology is modulated by the autonomic nervous system (ANS) consisting of sympathetic and parasympathetic nerves (Herring et al., 2019). An imbalance of the autonomic innervation may induce an inhomogeneous conduction and distribution of refractoriness promoting susceptibility to arrhythmia (Shen and Zipes, 2014). Autonomic stimulation (bilateral either sympathetic or parasympathetic) of isolated rabbit hearts for example shows spatial dispersion of repolarization (nonuniformity of repolarization) and heterogenous APD, which contributes to arrhythmia vulnerability (Mantravadi et al., 2007). In a diabetic rat model, increased heterogeneity of sympathetic nerves and defected parasympathetic nerves leads to decreased atrial ERP and increased incidence of AF under sympathetic nerve stimulation (Otake et al., 2009).

## 4 Cardiac Macrophages Mediate Arrhythmogenesis by Promoting Cardiac Remodeling

**FIGURE 2:**
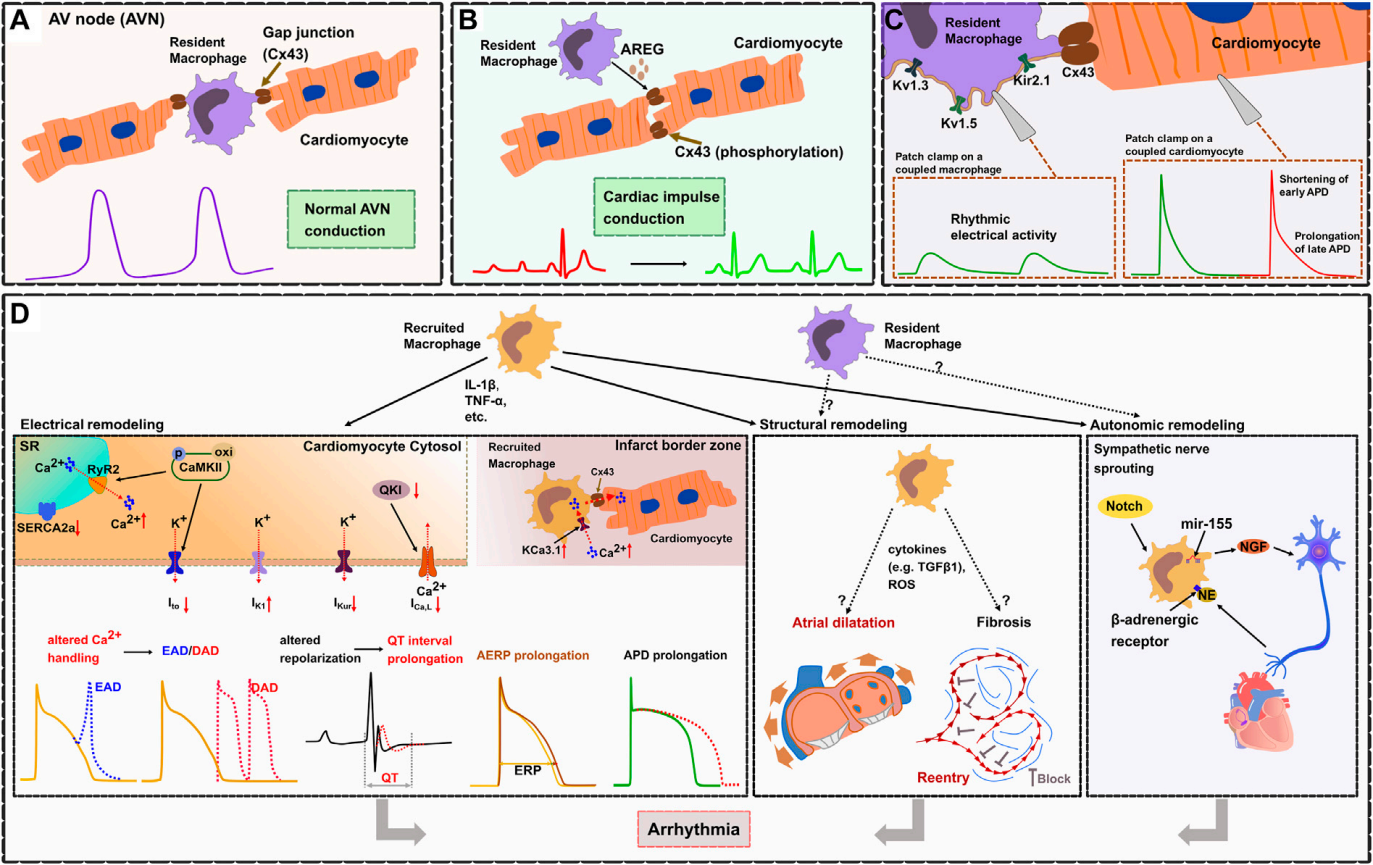
Cardiac macrophages in electrophysiology and arrhythmogenesis. **(A)**. In the healthy heart, cardiac resident macrophages are functionally linked to cardiomyocytes through gap junctions (Cx43), thereby facilitating electrical conduction in the atrioventricular node (AVN). **(B)**. Macrophages prevent arrhythmias by regulating the phosphorylation of Cx43 *via* AREG (Amphiregulin). **(C)**. Resident cardiac macrophages express potassium channels (including Kv1.3, Kv1.5, and Kir2.1), Patch clamp experiments show that resident macrophages can depolarize coupled cardiomyocytes, shorten early APD and prolong late APD. **(D)**. Electrical remodeling (left frame). During inflammation, recruited macrophages produce cytokines (IL-1β, TNF-α) which affect ion currents and calcium homeostasis resulting in increased electrical vulnerability to arrhythmias. TNF-α causes abnormal SR Ca^2+^-ATPase (SERCA2a) function which reduces the SR Ca^2+^ uptake. IL-1β induces AP prolongation through a decrease in I_to_ current and an increased diastolic sarcoplasmic reticulum (SR) Ca^2+^ leak *via* ryanodine receptors (RyR2). This is promoted through CaMKII oxidation/phosphorylation causing cytosol Ca^2+^ overload leading to delayed afterdepolarizations (DAD). CaMKII inactivates I_to_ which contributes to the prolongation of APD and predisposes to early or delayed afterdepolarizations (EAD). IL-1β reduces I_Ca,L_ by inhibiting the expression of atrial quaking protein (QKI) facilitating atrial fibrillation. Cytokines (IL-1β, TNF-α, TGF-β, IL-6) lead to APD prolongation by altering potassium current densities (increased I_K1_ and reduced I_Kur_). Reduced atrial conduction velocity and AERP prolongation, also results in enhanced susceptibility for atrial fibrillation (AF). Within infarct border zones, upregulated potassium channel KCa3.1 in recruited macrophages facilitate Ca^2+^ influx into the macrophages. Elevated intracellular Ca^2+^ then flows from recruited macrophages to adjacent cardiomyocytes via Cx43, which causes APD prolongation of cardiomyocytes. Structural remodeling (middle frame). Recruited macrophages cause atrial dilatation and fibrosis probably by releasing cytokines (e.g., TGF-β1) or reactive oxygen species (ROS), but the exact mechanisms have not been fully elucidated. Atrial dilatation and formation of reentry subsequently results in enhanced susceptibility for AF. Autonomic remodeling (right frame). Recruited macrophages induce autonomic nerve sprouting by synthesizing nerve growth factor (NGF). Norepinephrine (NE) from sympathetic nerve endings can in turn activate *β*-adrenergic receptors on macrophages, which enhances the expression of NGF. Activation of Notch signaling and microRNA-155 expression in recruited macrophages also promotes sympathetic outgrowth.

It has been suggested that macrophages play an important role in arrhythmogenesis since they can produce a number of cytokines which have been linked to cardiac remodeling. However, only recently a few studies have been published that clearly demonstrate specific macrophage-dependent mechanisms regulating physiologic conduction (Hulsmans et al., 2017; Sugita et al., 2021; Simon-Chica et al., 2022) as well as electrical, structural, or autonomic remodeling leading to arrhythmias (Monnerat et al., 2016; Sun et al., 2016; Yin et al., 2016; Fei et al., 2019; Hu et al., 2019; Liu et al., 2019; Lubos et al., 2020; Lyu et al., 2020; Miyosawa et al., 2020; Zhang et al., 2020; Hiram et al., 2021; Zhang et al., 2021) (Figure 2).

### 4.1 Cardiac Macrophages Modulate Cell-To-Cell Coupling *via* Gap Junctions

Cardiomyocytes are electrically connected *via* gap junction channels (established by connexins) which is an essential prerequisite for electrical impulse propagation. Modifications of connexins such as phosphorylation or altered distribution of gap junctions result in reduced conduction velocity or altered pathways of conduction and anisotropy leading to a proarrhythmic substrate predisposing to reentry (Jongsma and Wilders, 2000; Dhein and Salameh, 2021).

Recently, Hulsmans et al. (2017) demonstrated that cardiac resident macrophages are enriched in the healthy AV node both in mouse (CD11b^+^CD64^+^CX_3_CR_1_^+^F4/80^+^Ly6C^low^) and human (CD68^+^CD163^+^) and that they are functionally coupled to cardiomyocytes *via* Cx43. They revealed that macrophages coupled to cardiomyocytes show spontaneous rhythmic depolarizations whereas cardiomyocytes coupled to AV nodal macrophages show an elevated resting membrane potential and a shortened action potential, which facilitates electrical conduction in the AV node. These findings were further supported by several *in vivo* models: 1) using an optogenetics approach they were able to specifically depolarize AV nodal macrophages *in situ* (transgenic expression of the fluorescently activated channel rhodopsin-2 specifically in macrophages, Cx_3_cr1^wt/CreER^ ChR2^wt/fl^; focused light on the exposed AV node region) which resulted in enhanced electrical conduction through the AV node illustrated by a higher number of conducted stimuli at the Wenckebach point. 2) In a transgenic mouse model with macrophage-specific knockdown of Cx43 (Cx_3_cr1^wt/CreER^ Cx43 ^fl/fl^) they demonstrated an increased Wenckebach cycle length and AV node refractory period indicating that disruption of the macrophage-cardiomyocyte interaction results in impaired electrical conduction in the AV node. These findings were further confirmed by 3) a transgenic mouse model that congenitally lacks macrophages (Csf1^op^). 4) Inducible macrophage depletion in CD11b^DTR^ mice finally showed progressive AV block within 1–2 days. All these findings illustrate that cardiac resident macrophages facilitate electrical conduction in the AV node by electrical coupling to cardiomyocytes *via* Cx43 (Hulsmans et al., 2017) (Figure 2A).

In a mouse model of right ventricular (RV) stress (pulmonary artery banding leading to RV pressure overload), cardiac macrophages protect the heart from arrhythmia and sudden cardiac death (SCD) by maintaining proper electrical conduction through gap junctions (Sugita et al., 2021). Depletion of macrophages in this model resulted in advanced heart block and lethal cardiac arrest indicating that macrophages play an essential role for survival. Further studies identified macrophage-derived amphiregulin (AREG) as key regulator of Cx43 phosphorylation and translocation in cardiomyocytes. AREG knockout mice showed reduced Cx43 phosphorylation and a pronounced Cx43 lateralization leading to arrhythmias and increased mortality after pulmonary artery banding, while bone marrow transplantation (wildtype mice serving as donors) or treatment with recombinant AREG restored Cx43 phosphorylation/localization and prevented SCD (Sugita et al., 2021) (Figure 2B).

A recent study by Simon-Chica and colleagues comprehensively characterized the electrophysiologic properties of murine cardiac resident (Cx_3_cr_1_^eYFP/+^) macrophages and reported a membrane resistance of 2.2 ± 0.1 GΩ, a capacitance of 18.3 ± 0.1 pF, and a resting membrane potential of -39.6 ± 0.3 mV (Simon-Chica et al., 2022) (Figure 2C). They revealed that resident macrophages express potassium channels including Kv1.3, Kv1.5, and Kir2.1 which establish several inward and outward rectifying currents. Furthermore, resident macrophages have been shown to express Cx43. Computer modeling demonstrates that resident macrophages can depolarize coupled cardiomyocytes and shorten APD. Besides a number of novel findings, the authors could independently confirm several observations such as the macrophages’ resting membrane potential, their Cx43 expression and their ability to shorten the APD of coupled cardiomyocytes which have been previously reported by Hulsmans et al. (Hulsmans et al., 2017).

In patients with arrhythmias after myocardial infarction (but not in patients without post-MI arrhythmias) an elevated number of recruited macrophages have been detected in the infarct border zone where they form gap junctions with adjacent cardiomyocytes, a finding that was confirmed in a mouse model of MI (Fei et al., 2019). Recruited macrophages couple to cardiomyocytes *via* Cx43 in border zones of murine infarcted hearts and show an upregulation of the potassium channel KCa3.1 (Fei et al., 2019). This facilitates Ca^2+^ influx and causes APD prolongation in connected cardiomyocytes which results from the intracellular Ca^2+^ flow from macrophages to cardiomyocytes through Cx43, demonstrating that recruited macrophages can be functionally linked to cardiomyocytes modulating cardiac conduction (Fei et al., 2019) (Figure 2D).

In mice with myocardial infarction macrophage-produced proinflammatory cytokine MMP-7 was shown to be able to process Cx43 at a C-terminus cleavage site. The degradation of ventricular Cx43 is linked with decreased conduction velocity and increased incidence of arrhythmia and SCD (Gutstein et al., 2001; Lindsey et al., 2006). IL-1β has also been shown to lead to defective excitation-contraction coupling and arrhythmogenesis by Cx43 degradation in the post-MI heart (De Jesus et al., 2017). In the border zone of a MI, myofibroblasts emerge from fibroblasts under the stimulation of IL-1β, which then further produce more IL-1β, and cause Cx43 downregulation and thereby abnormal conduction of cardiac impulse and formation of arrhythmogenesis substrate (Baum et al., 2012).

Altogether, there is strong evidence that cardiac macrophages can be functionally linked to cardiomyocytes, modulate the cardiomyocytes’ electrophysiologic properties and may thus be key players in arrhythmogenesis.

### 4.2 Cardiac Macrophages Induce Cardiac Electrical Remodeling

A growing body of evidence suggests that inflammatory processes are key factors in the pathophysiology of atrial fibrillation (AF) with several remodeling mechanisms being regulated by macrophages (Engelmann and Svendsen, 2005; Yamashita et al., 2010; Hu et al., 2015; Mitrofanova et al., 2016; Watson et al., 2020). In patients with AF increased levels of inflammatory markers (e.g., CRP, IL-6, IL-8, TNF-α etc.) and an elevated number of recruited macrophages (identified as CD68^+^ and CD14^++^CD16^−^ macrophages) have been described in left and right atria (Patel et al., 2010; Yamashita et al., 2010; He et al., 2016; Sun et al., 2016; Aguiar et al., 2019).

Further mechanistic studies in lipopolysaccharide (LPS)-treated mouse and canine models revealed that recruited macrophages induce electrical remodeling by secreting pro-inflammatory cytokines, including TNF-α, IL-1β, and IL-6 (Sun et al., 2016) (Figure 2D). In these models, IL-1β has been shown to inhibit the expression of atrial quaking protein (QKI), a RNA-binding protein that regulates RNA splicing and maintains RNA stability and has been shown to reduce CACNA1C (L-type calcium channel subunit) expression and *I*_Ca,L_ in atrial myocytes leading to AF (Tili et al., 2015; Sun et al., 2016).

Liu and colleagues induced myocardial infarction in rats and treated these rats with Fisetin, a flavonoid with proposed anti-inflammatory effects (Liu et al., 2019). After 4 weeks post-MI rats showed significant LA fibrosis, prolonged interatrial conduction time and atrial refractory periods as well as an increased inducibility of AF, accompanied by elevated numbers of recruited CD68^+^ macrophages and increased expression of IL-1β and TNF-α in LA indicating proarrhythmic structural and electrical remodeling together with an inflammatory response (Figure 2D). In rats treated with Fisetin, macrophage recruitment to the heart, proarrhythmic remodeling and susceptibility for AF was significantly reduced suggesting a causal role for recruited cardiac macrophages in ischemia-related arrhythmogenesis (Liu et al., 2019).

Zhang and colleagues studied arrhythmogenesis in spontaneously hypertensive rats and could demonstrate that mechanisms mediated by recruited macrophages play an important role (Zhang et al., 2020). Hypertensive rats showed electrical and structural remodeling including APD prolongation, altered potassium current densities (increased *I*_K1_ and reduced *I*_Kur_), reduced expression of Cx43 as well as enlarged atria, enhanced atrial fibrosis and increased levels of reactive oxygen species, ultimately leading to an increased susceptibility for AF (Figure 2D). The authors proposed a macrophage-mediated mechanism due to elevated numbers of Mac2^+^ recruited macrophages in the atria of hypertensive rats and increased levels of common macrophage-derived cytokines such as TGF-β, IL-1β, IL-6, and TNF-α. CXCR2 inhibition blocked recruitment of macrophages, reversed all these effects and could finally prevent AF inducibility suggesting a macrophage-dependent mechanism (Zhang et al., 2020).

Inflammatory and macrophage-mediated arrhythmia mechanisms have also been shown for ventricular arrhythmias. Recent studies revealed that expression of *KCNN4* is upregulated in macrophages recruited to the heart after myocardial infarction. *KCNN4* encodes the intermediate conductance Ca^2+^-activated K^+^ channel KCa3.1, which can preserve the negative membrane potential required for sustained Ca^2+^ influx (Xu et al., 2017). As already mentioned in section 4.1, in the mouse MI model KCa3.1 activation in recruited macrophages facilitates Ca^2+^ influx and causes APD prolongation of cardiomyocytes which are connected to these macrophages *via* gap junctions, ultimately resulting in prolonged QTc duration and enhanced susceptibility to ventricular arrhythmias (Figure 2D) (Fei et al., 2019).

Diabetes mellitus is associated with an increased risk for arrhythmias (Kahn et al., 1987; El-Atat et al., 2004; Renner et al., 2020). For a long time, inflammation was proposed to play an important role in diabetic arrhythmogenesis, but only recently Monnerat and colleagues could demonstrate a specific macrophage-dependent mechanism (Figure 2D) (Monnerat et al., 2016). In a mouse model of diabetes mellitus, they showed that hyperglycemia activates the toll-like receptor 2 (TLR2) and the NLRP3 inflammasome in recruited cardiac MHC-II^high^ macrophages which in turn causes release of IL-1β. Elevated IL-1β leads to ventricular arrhythmias by prolongation of the ventricular action potential, reduction of the potassium current *I*_to_ and enhancement of a diastolic SR calcium leak which is mediated by increasing CaMKII oxidation/phosphorylation. Targeting the IL-1β axis by either inhibiting the IL-1β receptor or inhibiting the NLRP3 inflammasome was further shown as potential therapeutic approach protecting against arrhythmias (Monnerat et al., 2016).

### 4.3 Cardiac Macrophages Mediate Structural Remodeling

Studies on cardiac remodeling after tissue injury (e.g. myocardial infarction) revealed that macrophages play a pivotal role in cardiac remodeling as they may have both profibrotic and antifibrotic functions (Wynn and Barron, 2010; Liao et al., 2018; Revelo et al., 2021; Dobaczewski et al., 2011). CD68^+^ or CD11c^+^ macrophages infiltrate the epicardial adipose tissue of AF patients and are associated with atrial fibrotic remodeling leading to a proarrhythmic substrate for AF (Abe et al., 2018). As fibrosis is one of the hallmarks of arrhythmogenesis, it seems obvious that macrophages may play an important role in proarrhythmic structural remodeling, but specific studies are rare (Figure 2D).

The crosstalk between macrophages and cardiac fibroblasts regulates the balance of cardiac fibrosis (Van Linthout et al., 2014). Ly6C^high^ macrophages release anti-fibrotic cytokines like Osm to inhibit the conversion of fibroblasts to myofibroblasts. Ly6C^low^ macrophages in contrast produce profibrotic cytokines such as TGF-β1 and IL-10 to promote fibrosis (Weber et al., 2013; Abe et al., 2019). In turn, cardiac fibroblasts, as a major source of cytokines, can instruct resident macrophages to recruit other immune cells (monocytes, neutrophils) (Van Linthout et al., 2014). The cytokine secretion (IL-6, TGF-β1) of cardiac fibroblasts relies on the presence of macrophages in an *in vitro* co-culture model (Ma et al., 2012). Alvarez et al. (2011) explored the relationship between macrophages and fibroblasts in association with congenital heart block (CHB). They found that human fetal cardiac fibroblasts secreted TGF-β under the stimulation of macrophage-produced endothelin-1 in an *in vitro* experiment, and endothelin-1 presented in the septal region in areas of calcification and fibrosis in two fetal hearts of CHB. Furthermore, cardiac fibroblasts can influence cardiac function through direct (through gap junctions or membrane nanotubes) and indirect (paracrine signaling) effects on cardiomyocytes (Camelliti et al., 2004; Miragoli et al., 2006; Quinn et al., 2016).

In AF patients without concomitant heart failure, elevated numbers of macrophages (CD163^+^) were detected in right atrial appendages which was associated with increased atrial gene expression of procollagen and B-type natriuretic peptide (BNP) and atrial fibrosis (Watson et al., 2020). Although sample size was small (RA tissue from 10 patients with AF and 27 patients without AF) this study indicates a direct link between cardiac macrophages, structural remodeling and AF.

In another small study, elevated numbers of recruited CCR2^+^ macrophages have been observed in left atrial appendages obtained from AF patients (Miyosawa et al., 2020). Macrophage numbers were higher in patients with left atrial dilatation compared to patients without LA dilatation, indicating an association between increased macrophage numbers and atrial structural remodeling. In a larger cohort (83 patients with AF and normal LA diameter, 78 patients with AF and LA dilatation, 22 patients without AF) the authors further studied circulating monocytes. In AF patients the numbers of monocytes were significantly lower compared to patients without AF but among AF groups total numbers or proportion of monocyte subsets were not different. However, in patients with LA dilatation monocytes expressed higher levels of CCR2 and exhibited an enhanced migratory activity *in vitro*. Although this study is also purely descriptive, investigated a small number of patients and lacks a comprehensive analysis of structural remodeling (e.g. atrial fibrosis) it is another hint towards a direct role of recruited cardiac macrophages in proarrhythmic structural remodeling (Miyosawa et al., 2020).

In rats, monocrotaline-induced pulmonary hypertension causes proarrhythmic atrial remodeling including reduced atrial conduction velocity, atrial dilatation and fibrosis which subsequently results in enhanced susceptibility for AF (Hiram et al., 2021) (Figure 2D). The authors proposed a macrophage-related mechanism since an elevated number of recruited CD68^+^ macrophages was observed in the right atrium. Treatment with resolvin-D1 significantly attenuated proarrhythmic remodeling and reduced the inducibility of AF which was accompanied by significant reduction of CD68^+^ macrophages and an increase of CD206^+^ macrophages in the heart (Hiram et al., 2021).

Several independent studies performed in rat models provide some more mechanistic data which support a clear link between cardiac macrophages and proarrhythmic remodeling (Figure 2D). As these studies by Yun-Long Zhang et al. (2020), Liang Liu et al. (2019) indicated macrophage-related effects both on structural and electrical remodeling, they are already discussed in section 4.2.

Arrhythmogenic cardiomyopathy is an inherited disease characterized by progressive structural remodeling ultimately leading to arrhythmias and SCD (Elliott et al., 2019). In mice, inflammation has been identified as a major mechanism of macrophage recruitment to the heart (Lubos et al., 2020). Histologic analyses revealed that CD11b^+^CD206^+^F4/80^+^ macrophages accumulate and persist in fibrotic regions/scars over several weeks and that the macrophage populations include both proinflammatory (expressing MMP12) and reparative (expressing osteopontin) macrophages. Although this work illustrates the association of macrophages with fibrotic remodeling, potential conclusions on arrhythmogenesis must be drawn with caution as 1) there was no direct evidence for arrhythmias in these mice, 2) direct macrophage function/effects were not investigated, and 3) a direct causal role for macrophages remains elusive.

All these studies mentioned above demonstrate a potential role for recruited, i.e., monocyte-derived cardiac macrophages in proarrhythmic remodeling. In heart failure induced by aortic constriction in mice (pressure overload TAC model) it has been indicated that cardiac resident (CCR2^−^) macrophages and recruited monocyte-derived (CCR2^+^Ly6C^high^) macrophages may have distinct and at least partly antagonistic effects (Liao et al., 2018; Revelo et al., 2021) suggesting that different macrophage populations in the heart may also differentially affect proarrhythmic remodeling.

### 4.4 Macrophages Mediate Autonomic Nerve Remodeling

The heart is innervated both by sympathetic and parasympathetic nerves and alterations in this innervation—the so-called autonomic remodeling—have been demonstrated to play important roles in arrhythmogenesis (Cao et al., 2000; Stavrakis et al., 2020). Macrophages are able to synthesize nerve growth factor (NGF), an essential protein for promoting sympathetic nerve sprouting (Brown et al., 1991; Wernli et al., 2009) indicating a potential role for macrophages in autonomic remodeling.

This is further supported by the presence of CD68^+^ macrophages in stellate ganglia from patients with Long QT syndrome (LQTS) or catecholaminergic polymorphic VT (CPVT) where the autonomic innervation has been identified as key factor in arrhythmogenesis (Rizzo et al., 2014). Studies in a rat heart failure model revealed that local depletion of macrophages in stellate ganglia attenuate cardiac sympathetic overactivation and susceptibility to ventricular arrhythmias (Zhang et al., 2021). This is due to reduced levels of proinflammatory cytokines (TNF-α and IL-1β) and reduced N-type Ca^2+^ currents as well as excitability of cardiac sympathetic neurons (Zhang et al., 2021). Norepinephrine (NE) from sympathetic nerve endings can in turn activate *β*-adrenergic receptors on macrophages, which enhances the expression of NGF and establishes a vicious circle of sympathetic nerve remodeling, thereby aggravating electrophysiological heterogeneity and increasing the risk for ventricular arrhythmias (Lyu et al., 2020).

Notch signaling is an important pattern receptor for myeloid cell differentiation and macrophage activation (Monsalve et al., 2006). In post-MI rat hearts, the activation of Notch signaling predominantly promotes polarization of recruited macrophages toward the proinflammatory phenotype in the infarcted border zone as well as NGF expression, which then stimulates sympathetic outgrowth (Yin et al., 2016). Inhibition of Notch in contrast induces a phenotype switch from proinflammatory to reparative macrophages and decreases NGF expression, consequently ameliorating sympathetic nerve sprouting (Yin et al., 2016). The macrophage-derived microRNA-155 has been shown to suppress genes related to neural stem cell self-renewal by downregulation of cytokine signaling 1 (Scos1) (Zingale et al., 2021). Inhibition of microRNA-155 expression in recruited macrophages in a MI mouse model displays decreased density of tyrosine hydroxylase and GAP43 (neuromodulin) positive nerve fibers in myocardial tissue, which was associated with reduced ventricular arrhythmias (Hu et al., 2019) (Figure 2D).

In sum, a number of studies indicate that recruited macrophages may play an important role in arrhythmogenesis by regulating autonomic remodeling after myocardial injury.

## 5 Future Challenges: Studying Macrophages in Arrhythmogenesis Following the Translational Road From Rodents to Humans

**FIGURE 3:**
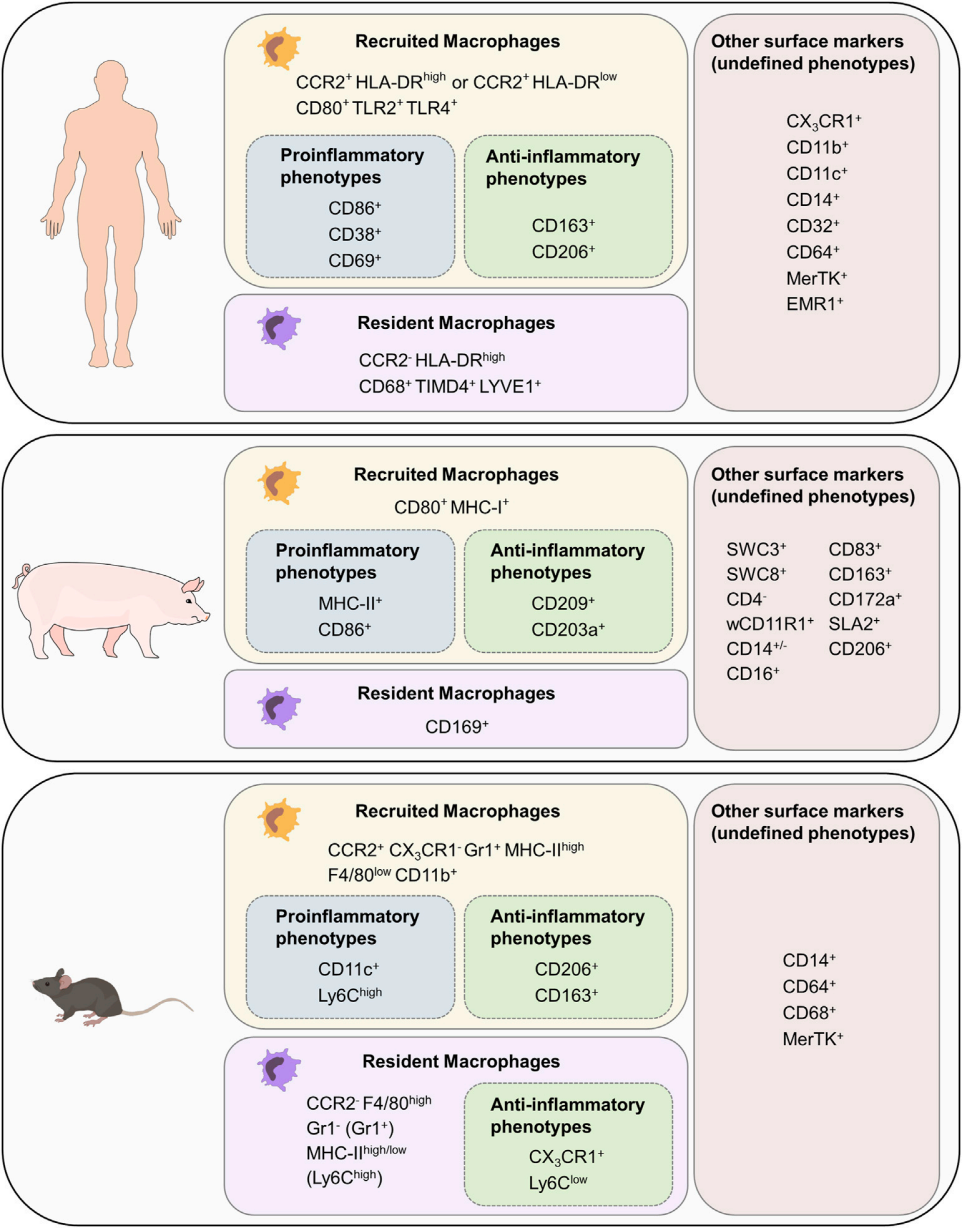
Cardiac macrophage surface markers in mouse, pig and human. List of surface markers for recruited and resident macrophages as well as markers for proinflammatory and anti-inflammatory phenotypes. Surface markers with unknown association to function have been grouped as “other surface markers (undefined phenotypes). F4/80 in mouse is the homolog for EMR in humans.

To investigate the complex role of macrophages in electrophysiology and arrhythmogenesis, delicately designed *in vivo* experiments are required. Model organisms vary in regard to anatomy, physiology, electrophysiological properties, immunology, genetic background, availability, costs, practical, and ethical considerations (Clauss et al., 2019; Schuttler et al., 2020). All species used in research have distinct advantages and disadvantages and every species might be the ideal one for a specific research question. Since mouse models are widely available at different genetic backgrounds with various genetic modifications and manifold options for disease modeling they are highly suitable and widely used for initial investigations both in electrophysiology and immunology research. However, compared to humans mice have notable differences regarding both electrophysiology and immunology, which limits the transferability of findings obtained in mouse models to a clinical setting in humans. Thus, suitable so-called preclinical models in large animals are necessary to validate findings obtained in mouse models prior to clinical application. The most widely used large animal species include sheep, dogs and pigs. In regard to electrophysiology research we have proposed a ”practical trio” including mice, rabbits and pigs as these species are most suitable for the majority of researchers in the field (Clauss et al., 2019). Furthermore, such a “practical trio” establishes a translational concept paving the way from early *in vitro* studies for screening, over initial *in vivo* validation in mice and further mechanistic studies in rabbits to confirmation and preclinical testing in close-to-human pig models prior to clinical application in human patients (Clauss et al., 2019). Continuing our proposed concept, we will discuss how cardiac macrophage populations differ between mice, pigs, and humans, by which surface markers they can be identified (Figure 3), and finally demonstrate that the pig is a suitable large animal species to study cardiac macrophages in the context of arrhythmias.

As described in section 1, murine resident macrophages derived from embryonic/fetal precursors and recruited macrophages derived from bone-marrow monocytes can be distinguished based on their CCR2 and CX_3_CR_1_ expression (Pinto et al., 2012; Bajpai et al., 2019). Resident macrophages (CCR2^-^ CX_3_CR_1_^+^) can be further grouped into two numerically dominant Ly6C^low^Gr1^−^MHC-II^high^ and Ly6C^low^Gr1^−^MHC-II^low^ subsets, and the Ly6C^high^Gr1^+^ subset. Furthermore, resident macrophages can be identified as F4/80^high^, whereas recruited macrophages (CCR2^+^CX_3_CR_1_^−^) are F4/80^low^CD11b^high^. Recruited macrophages with a proinflammatory phenotype also express CD11c, whereas those with a reparative phenotype can be identified as CD163^+^CD206^+^ (Zhu et al., 2017; Ma et al., 2018). Other classic murine macrophage markers include CD14, CD64, CD68, and MerTK (Gautier et al., 2012).

Though rabbits are a valued model in electrophysiology research, cardiac macrophages in rabbits have been poorly characterized so far. Rabbit macrophages can be polarized into proinflammatory (MHC-II^high^) and reparative (CD206^+^) phenotypes using human recombinant GM-CSF and M-CSF, respectively (Yamane and Leung, 2016). Rabbit macrophages do not express the tissue macrophage marker CD68, but express RAM11, a widely used macrophage marker in rabbits (Lis et al., 2010). CD14, CD163, CD200 and Arginase-1 can also be used to further group rabbit macrophages (Sanz et al., 2007; Hoemann et al., 2010; Akkaya et al., 2016; Desando et al., 2019). Rabbits, as they are still easy to house, can be used to fill the gap between rodents and large animals.

Human macrophages are grouped into three major subpopulations depending on the cell surface markers CCR2 and HLA-DR. Since attraction of monocytes into tissue depends on CCR2 signaling, recruited macrophage populations can be identified as CCR2^+^HLA-DR^low^ and CCR2^+^HLA-DR^high^ (Svedberg and Guilliams, 2018). Resident cardiac macrophages, however, are CCR2^−^HLA-DR^high^ (Bajpai et al., 2018; Sansonetti et al., 2020). Both CCR2^+^ and CCR2^-^ macrophages express common markers of monocytes and macrophages including CX_3_CR_1_, CD11b, CD11c, CD14, CD32, CD64, CD86, MerTK, and EMR1 (Bajpai et al., 2018). The majority of CD68^+^ cells represent CCR2^-^ resident macrophages which also express TIMD4 and LYVE1 (Dick et al., 2019). To distinguish functional phenotypes of recruited macrophages many additional markers can be used: human proinflammatory macrophage markers include CD38, CD69, CD80, TLR2, and TLR4, whereas human anti-inflammatory macrophage markers include CD163 and CD206 (Singleton et al., 2016; Yunna et al., 2020; Kim et al., 2021).

Porcine macrophages have not been characterized to the same extent, making it challenging to identify monocyte/macrophage lineage cells based on their surface markers (Piriou-Guzylack and Salmon, 2008; Ezquerra et al., 2009; Mair et al., 2014; Dawson and Lunney, 2018; Nikovics and Favier, 2021). In blood cell maturation, two cell lineages can be distinguished from each other: The lymphoid lineage leads to T-, B- and natural killer (NK) cells. Granulocytes, monocytes, macrophages and polymorphonuclear cells (PMCs) are part of the myeloid cell lineage (Kondo, 2010). Porcine memory helper T cells present as CD4^+^CD8^+^cells. B cells can be identified with the marker CD21, T cells can be furthermore distinguished with being CD3e^+^CD56^+^. NK cells express the marker CD56 (Piriou-Guzylack and Salmon, 2008). As these markers are not present in the myeloid lineage, spotting and excluding cells of the lymphoid lineage eases identification of macrophages. All cells with origin from the myeloid progenitor line express high levels of the swine workshop cluster SWC3, which makes it an ideal marker for myeloid cells (Summerfield and McCullough, 1997). Porcine PMCs are described as SCW1^+^SWC3^+^SWC8^+^SWC9^−^CD14^low^CD163^-^. Dendritic cells can be identified as SWC3^+^WC1^+^wCD11R1^−^CD16^+^CD163^-^ cells (Piriou-Guzylack and Salmon, 2008). CD203a and SWC8 function as potential identification markers for myeloid differentiation: when monocytes develop into macrophages, they express more CD203a. SWC8 can be found on pig PMCs in the blood and on macrophages (Piriou-Guzylack and Salmon, 2008). CD11b is a commonly used marker for human monocytes and macrophages. The anti-human CD11b antibody reacts with the porcine wCD11R1 but in contrast wCD11R1 is not expressed on porcine monocytes or alveolar macrophages. CD14, like in humans, is highly expressed on porcine monocytes, is also present on a low level on tissue macrophages and has a low expression level on porcine granulocytes (Piriou-Guzylack and Salmon, 2008; Ezquerra et al., 2009). CD172a serves as a marker for bone marrow cells, which origin in the myeloid/monocyte/macrophage lineage, and is therefore present on macrophages, neutrophils and dendritic cells. CD16 is expressed on porcine NK cells, monocytes and macrophages (Ezquerra et al., 2009). In sum, porcine monocytes can be described as SWC1^+^SWC3^high^CD11a^high^wCD11r1^low/−^CD14^high^MHC-II^+/−^SWC8^−^SWC9^−^CD163^+/−^CD49e^high^CD49d^high^ cells (Piriou-Guzylack and Salmon, 2008). F4/80 encoded by EMR1, which was later renamed to Adgre1, is a commonly used marker for tissue-resident macrophages in mice. Anti-porcine Adgre1 monoclonal antibodies detect monocytes and granulocytes in bone marrow and blood as well as porcine tissue macrophages (Waddell et al., 2018). CD163 has an especially high expression on porcine monocytes and macrophages which makes it an ideal marker for these cells (Mair et al., 2014; Dawson and Lunney, 2018), whereas CD169 is only expressed on tissue macrophages. MHC-II can be used as a marker for porcine recruited macrophages but is more present on B lymphocytes, microglia and dendritic cells. SLA2 is expressed on dendritic cells, B cells, monocytes, macrophages and also on some T cell subpopulations (Dawson and Lunney, 2018). CD80 is an established porcine macrophage marker, but cannot distinguish between classic proinflammatory and alternatively activated anti-inflammatory macrophages as it is expressed on both cell types (Nikovics and Favier, 2021). To further distinguish different porcine macrophage subsets, proinflammatory macrophages can be identified as CD86^+^CD203a^+^ (Singleton et al., 2016) whereas anti-inflammatory macrophages can present as MHC-I^+^CD86^+^ cells (Carta et al., 2021).

When choosing a suitable model for arrythmia studies, many aspects have to be taken into consideration. There are notable differences between species in regard to electrophysiology, including different expression and distribution of ion channel subunits in the heart, or action potential morphology and duration (Kaese and Verheule, 2012; Kaese et al., 2013; Clauss et al., 2019; Schuttler et al., 2020). Compared to the human AP, the murine AP is characterized by a faster repolarization phase, no prominent plateau phase, and an overall more negative membrane potential (Kaese et al., 2013; Boukens et al., 2014; Clauss et al., 2019; Schuttler et al., 2020). In the mouse, *I*_to_ and *I*_Kur_ play important roles in ventricular repolarization, whereas in humans *I*_Kr_ and *I*_Ks_ are the main repolarization currents (Joukar, 2021). Further differences include NCX which contributes only around 7% to Ca^2+^ clearance (in humans: around 28%–29%) (Edwards and Louch, 2017), the *I*_Ca,L_ current causing the lack of an obvious plateau phase (Kaese and Verheule, 2012), and *I*_K1_ which is less important for repolarization in mice (Boukens et al., 2014).

Pigs have been increasingly used in cardiovascular research as they closely resemble the human cardiac anatomy and (electro)physiology (Verma et al., 2011). Furthermore, the porcine immune system is very similar to the human immune system, especially compared to mice (Mair et al., 2014; Pabst, 2020). Clinical routine techniques and equipment used for humans can easily be applied to pigs, a large number of disease models have been established and well characterized (Schuttler et al., 2022), and targeted genetic modification has become feasible with recent technological advances (Park et al., 2015; Langin et al., 2018; Renner et al., 2020; Riedel et al., 2020; Stirm et al., 2021). Moreover, pigs present action potentials and electrophysiological properties similar to humans as they express the same major ion currents (except *I*_to_) (Kaese et al., 2013; Piktel and Wilson, 2019). Major differences exist in regard to the phase 1 notch in ventricular cardiomyocytes resulting from *I*_to2_ (Ca^2+^-activated Cl^−^, also called *I*_Ca,Cl_) current in pigs and from *I*_to_ current in humans (Li et al., 2003).

Although more research on specific porcine macrophage subsets is warranted to allow a more precise classification, pigs have several obvious advantages over other species especially regarding the resemblance to human electrophysiology and immunology, which make them an ideal choice as large animal species for studying cardiac macrophages in arrhythmogenesis.

## 6 Conclusion

Inflammation is thought to crucially contribute to the development of an arrhythmogenic substrate. Emerging data underlines vital roles of distinct cardiac macrophage subsets for regulating proarrhythmic electrical, structural, or autonomic remodeling. Given their remarkable plasticity, multiple origins and phenotypes, further analysis of the functionality of macrophage subsets in arrhythmogenesis is clearly necessary. A better understanding of arrhythmia mechanisms will reveal new potential therapeutic targets allowing the development of innovative therapeutic strategies for patients suffering from arrhythmias. To achieve this goal, initial findings on macrophage-mediated arrhythmia mechanisms obtained in rodent models need to be validated in preclinical close-to-human large animal models prior to clinical application. As they share close similarities with humans regarding cardiovascular anatomy, electrophysiology and immunology we propose pigs as suitable large animal species for translational research on cardiac macrophages and their role in arrhythmias.
